# High-throughput sequencing of black pepper root transcriptome

**DOI:** 10.1186/1471-2229-12-168

**Published:** 2012-09-17

**Authors:** Sheila MC Gordo, Daniel G Pinheiro, Edith CO Moreira, Simone M Rodrigues, Marli C Poltronieri, Oriel F de Lemos, Israel Tojal da Silva, Rommel TJ Ramos, Artur Silva, Horacio Schneider, Wilson A Silva, Iracilda Sampaio, Sylvain Darnet

**Affiliations:** 1 Genetics and Molecular Biology Laboratory, Coastal Studies Institute, Bragança Campus, Universidade Federal do Pará, Bragança, PA, 68.600-000, Brazil; 2 Departamento de Genética, Faculdade de Medicina de Ribeirão Preto, Universidade de São Paulo, Centro Regional de Hemoterapia de Ribeirão Preto, Rua Tenente Catão Roxo, 2501, Ribeirão Preto, SP 14051-140, Brazil; 3 EMBRAPA Amazônia Oriental, Trav. Dr. Enéas Pinheiro s/nº, Caixa Postal 48, Belém, PA 66095-100, Brazil; 4 Instituto de Ciências Biológicas, Universidade Federal do Pará, Campus Universitário do Guamá, Rua Augusto Corrêa, nº1, Belém, PA 66075-110, Brazil

## Abstract

**Background:**

Black pepper (*Piper nigrum* L.) is one of the most popular spices in the world. It is used in cooking and the preservation of food and even has medicinal properties. Losses in production from disease are a major limitation in the culture of this crop. The major diseases are root rot and foot rot, which are results of root infection by *Fusarium solani* and *Phytophtora capsici*, respectively. Understanding the molecular interaction between the pathogens and the host’s root region is important for obtaining resistant cultivars by biotechnological breeding. Genetic and molecular data for this species, though, are limited. In this paper, RNA-Seq technology has been employed, for the first time, to describe the root transcriptome of black pepper.

**Results:**

The root transcriptome of black pepper was sequenced by the NGS SOLiD platform and assembled using the multiple-k method. Blast2Go and orthoMCL methods were used to annotate 10338 unigenes. The 4472 predicted proteins showed about 52% homology with the *Arabidopsis* proteome. Two root proteomes identified 615 proteins, which seem to define the plant’s root pattern. Simple-sequence repeats were identified that may be useful in studies of genetic diversity and may have applications in biotechnology and ecology.

**Conclusions:**

This dataset of 10338 unigenes is crucially important for the biotechnological breeding of black pepper and the ecogenomics of the Magnoliids, a major group of basal angiosperms.

## Background

Black pepper (*Piper nigrum*) is one of the most popular and oldest spices in the world, with culinary uses and more recently as a food preservative [1]. Due its medicinal properties, it is used in traditional medicine for its antioxidant, anti-inflammatory and anticancer properties [2,3]. This species is the second most widely traded spice in the world, accounting for a value of 929 million US dollars from a production of 445900 tons in 2009 [4].

*P. nigrum* is a perennial, climbing vine indigenous to the southwestern region of India. Its production is limited to rainy tropical regions such as those found in Asia and South America. Tropical climates favor the development of diseases, and crop losses due to pests and diseases are a major constraint in the production of black pepper [1]. Resistance against the two major pathogens, *Phytophtora capsici* and *Fusarium solani* f. sp*. piperis*, has not been achieved by classical breeding of the germplasm of cultivated black pepper and remains a major challenge that may be amenable to improvement using plant biotechnology [1]. Despite the agricultural and economic importance of black pepper, knowledge of its genetics is presently very limited [5].

For non-model plants such as black pepper with little or no molecular information available, next-generation sequencing (NGS) technologies offer a great opportunity for the rapid access to genetic information [6-9]. In plant biology, due to large sizes of genomes and high levels of ploidy, the characterization of transcriptomes is a powerful tool for the identification of proteins. RNA-seq can sequence, in an extremely high-throughput and quantitative manner, the transcriptome of an organism or tissue [9]. Next-generation sequencing technologies generate large numbers of reads with high sampling rates of cDNA libraries, providing a deeper and more complete view of transcriptomes [8]. This transcriptome-wide description of RNA sequences identifies the coding sequence, single nucleotide polymorphisms (SNPs), other polymorphisms, splicing variants and relative levels of expression by counting reads, a method known as digital gene expression (DGE) [9,10].

**Figure 1:**
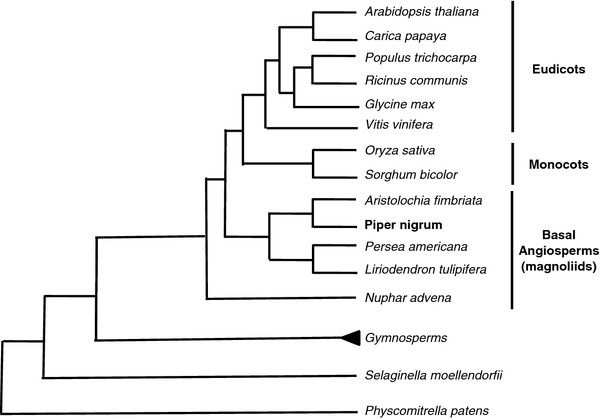
**Phylogenetic position of* Piper nigrum *with others plants group.**

An RNA-seq analysis of the black pepper transcriptome can greatly impact our knowledge of this species. Black pepper belongs to the Piperaceae family of the Magnoliid subclass, know as basal angiosperms, a large and important family for which little genomic data is available (Figure 1). Transcriptomic data could have many ecological and biotechnological repercussions, from exploring and characterizing the evolution of this family, to defining markers such as microsatellites that are useful in breeding programs, to establishing a catalog of expressed genes related to plant defense and metabolism.

In this work, we used RNA-seq technology to analyze the transcriptome of black pepper. About 71 millions reads were generated and 22363 *de novo* assembled transcripts were obtained from tissue of the stem-root. The transcripts were functionally annotated and represent the first sequences derived from the transcriptome of *P. nigrum*. About 257 new simple sequence repeats (SSRs) were described. These results, the first dataset of sequences of the Magnoliid group, will open new perspectives for studies of diversity and ecology and will benefit breeding and genomic programs aiming to understand plant-pathogen interactions in the root, the organ that is central in the process of infection by *F. solani* and *P. capsici*, the two major pathogens of black pepper.

## Results

### Sequencing results

The dataset of raw reads was deposited in NCBI database under SRA047721 accession number and contains about 71878419 reads 50 bp in length totalling 3.5 Gbp (Table 1). Pre-processing was performed with high stringency; reads with quality values (QVs) below 20 in windows averaging 3 bp and bases with QVs below 18 were trimmed. Reads shorter than 30 bp after pre-processing were excluded. After removing redundant reads, filtering reads of low quality, trimming low-quality ends and removing adapters, the size of the dataset was 13300000 millions reads, representing about 665 Mbp. Additional file 1: Figure S1 shows the QVs of the filtered read dataset as monitored by read position using FASTQC [11]. The average QV varies from 29 to 22 (base call accuracy of 99.37-99.85%), with a slow decrease along the read length. No bases with QVs under eight (base call accuracy under 85%) were observed, and more than 75% of bases had a QV over 12 (base call accuracy over 93.7%). Additional file 2: Figure S2 presents the length distribution of reads; 83% of reads have a length over 45 bp after trimming and filtering. 

**Table 1:** Overview of sequencing and assembly

**Raw data**	
Total number of reads	71878419
Number of unique high-quality reads	13300000
Number of contigs obtained with Velvet/Oases/STM^-^	22363
Unigenes dataset	
Unigenes number	10338
Total size (bp)	1787600
Largest transcript length (bp)	1314
N50 length (bp)	168
No ATGC characters	18
Average coverage per base	62
Predicted proteins	
Predicted CDS (partial/complete)	4472
Gene expression	
Reads mapped in transcripts	71148200
Most expressed transcript (rpkm)	68250

bp: base pair; rpkm: read coverage normalized per million mapped nucleotides and the length of unigene.

#### *De novo* transcriptome assembly

**Figure 2:**
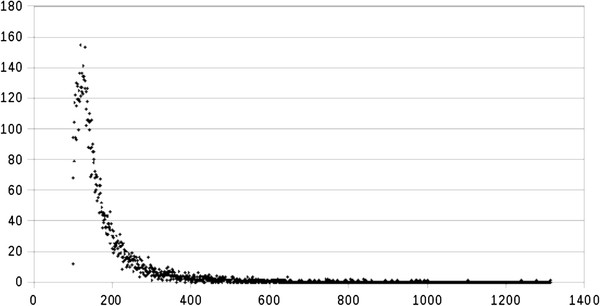
**Comparison of* de novo *assembly for different k-mers.** The bars indicate the number of contigs generated, and the hashed portions represent the percent of contigs that have homology in the databank of non-redundant proteins using BlastX. The lines indicate the N50 length (triangle) and the average length (lozenge) in bp. X-axis: k-mer number; Y-axis: read count.

The assembly of the transcriptome from the stem-root of black pepper was performed using the method of additive multiple-k described by Surget-Groba *et al*. [12]. The first step of this method is to assemble the reads using single k-mers varying from 19 to 43 bases. Figure 2 presents the results of assembly for each k-mer. The numbers of contigs decrease from 6847 (k-mer = 19 bases) to 22 (k-mer = 43 bases). The N50 and average length of contigs inversely increase from 166 bp to 214 bp and 151 bp to 236 bp, respectively, from k-mers of 19 to 43 bases in length (Figure 2). Thus, the average length of contigs is greater from larger k-mers, but the number of contigs generated is lower. The second step of the additive multiple-k method is to merge the assemblies using different k-mers, removing redundant contigs. This method has the advantage to detect low expressed genes and improve the assembly of long contigs. To identify possible isoforms of transcripts, the assembly was performed using the Oases script, which clusters similar contigs into small groups to derive isoforms of transcripts. The STM^-^ methods was used, considering the *Aristolochia fimbriata* predicted proteome as reference to generate scaffolds. 

A final dataset of 22363 contigs was obtained, corresponding to 3.8 Mbp. After contigs assembly, the contigs dataset was filtered using SeqClean tool and were removed 1357 contigs with rDNA sequences, 1640 with mitochondrial, 539 with chloroplastidial and 154 plant DNA repeats sequences (http://seqclean.sourceforge.net/). All contigs with a length inferior to 100 bp were excluded.

**Figure 3:**
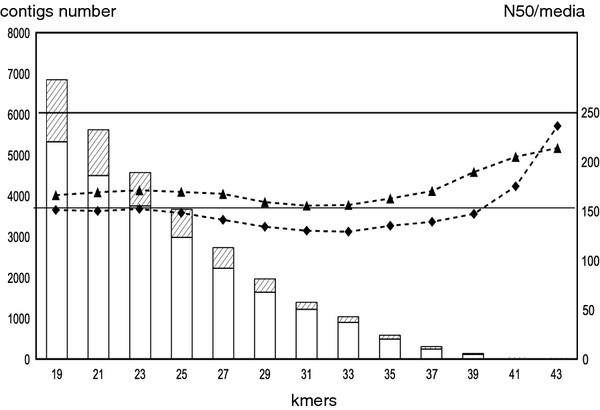
**Size distribution of the transcripts from 2-GS sequencing from the black pepper root.** Y-axis: count number; X-axis: size in bp.

Following the cleanup step, iAssembler tool was used to cluster and assembly contigs to obtain unigene sequences [13]. Finally, 10338 unigenes were identified from contigs dataset. The largest unigene is 1314 bp in length, and the N50 length is 168 bp (Table 1, Figure 3). 

**Figure 4:**
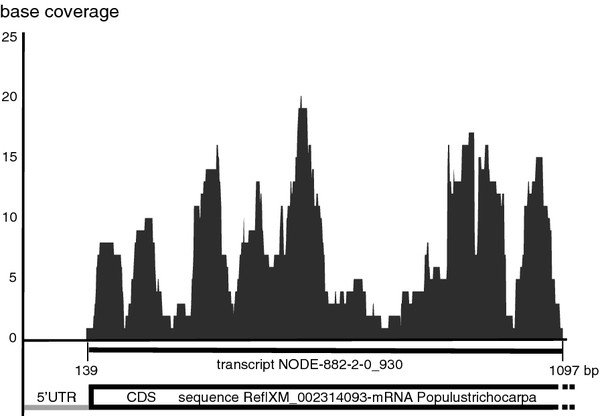
**Coverage per base for the NODE-882-2-0 transcript.** The coverage for each base. The most similar mRNA sequence identified in Genbank is reported above (Ref|XM_002314093). CDS = CoDing Sequence. Y-axis: base coverage; X-axis: base position in transcript.

The average read coverage is 211 reads per contig, and the highest coverage is 46239 reads for one contig, corresponding to 68250 in rpkm unit. The average coverage per base is 62x, which indicates that, on average, each base of the contigs was sequenced 62-fold. For example, the base coverage of contig NODE-882-2-0_930, one the longest of the dataset, was plotted, and the coverage per base varied from one to 22 along the 959 bp (Figure 4).

### Functional annotation of the transcriptome

The number of predicted partial or complete coding sequences (CDS) was 4472, representing about 44% of the total transcripts. An ncRNA search of the NOCODE databank [14] indicated that only 32 reads had significant homology with non-coding RNAs, with putative regulatory functions. About 52% of predicted proteins had a significant homology (limit of 1e^–05^) with *Arabidopsis thaliana* proteins sequences in the NCBI database (Table 2). The species with the highest hit rates were *Populus trichocarpa* with 54.38%, *Aristolochia fimbriata* with 54.02 % and *Vitis vinifera* with 53.93% of hits. The percentage of sequences from our dataset showing homology (1e^-5^) with proteomes of five model plants is reported in Table 2. The homology varied from 50% to 54%, with lower values obtained with monocots than with dicots. In Figure 4, the contig NODE-882-2-0_930 was aligned to the mRNA sequence of the putative pre-mRNA-splicing factor, RBM22/SLT11, from *Populus trichocarpa*. The *P. nigrum* contig and the *P. trichocarpa* mRNA show 77% identity in nucleotides along the putative CDS sequence. The corresponding protein sequences have an identity of 87% and a similarity of 96%, with a coverage of 94%, without gaps. 

**Table 2:** Percent of predicted proteins with homology to protein sequences from databases of other species

**Protein databases**	**% with homology**
*Arabidopsis thaliana*	51.57
*Glycine max*	53.23
*Vitis vinifera*	53.93
*Oryza sativa*	50.87
*Populus trichocarpa*	54.38
*Sorghum bicolor*	51.43
*Aristolochia fimbrata*	54.02

Predicted protein sequences were obtained used FrameD tool and BLASTP was performed with e-value of 1e^-05^.

**Figure 5:**
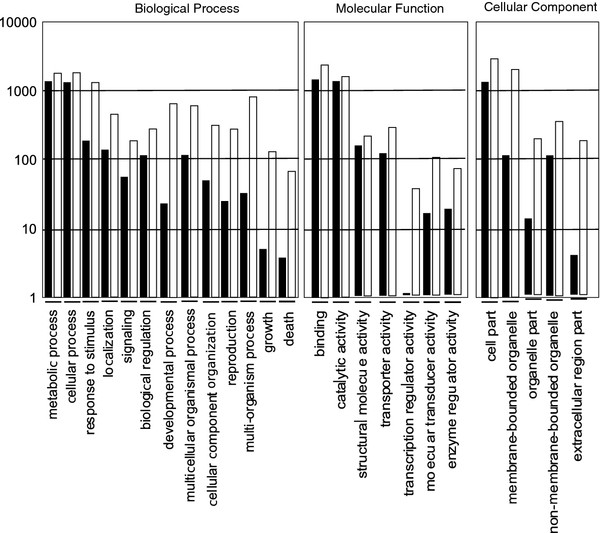
**Gene ontology classification of transcripts.** Black bar: *P. nigrum root library;* White bar: *A. thaliana root library.*

The functional annotation was performed using BLAST2GO results [15], and 3055 (29.5% of total unigenes) were functionally annotated with the gene ontology (GO) databank for *P. nigrum* root library and 9664 unigenes (73.3%) for one *A. thaliana* root library (AtGI-5336) used for comparison. The GO-SLIM annotations for black pepper root and one *Arabidopsis thaliana* root library are graphically presented in Figure 5. The biological processes with higher numbers of transcripts are in the categories “cellular process” and “metabolic process”, with respectively 1499 and 1328 unigenes. The “Molecular Function” classification reveals that the function “binding” is at a similar level as “catalytic activity”, with respectively 1712 and 1772 unigenes. The “cell part” annotations, as shown in Figure 5, indicate that a high proportion, about 39% of predicted CDS, should be localized in organelles. In Table 3 was reported the proteins number for the *A. thaliana* and *P. nigrum* root. Using the bioinformatics tools was predicted from cDNA sequences, 8004 protein sequences for *A. thaliana* root and 4472 for *P. nigrum* (Table 3). Using orthoMCL tool, were identified 3408 orthogroups and 37 paralogs for *A. thaliana* and 1017 and 159 for *P. nigrum*. The proportion of orthogroups/proteins number for each plant is different, 42.6% for *A. thaliana* and 22.7% for *P. nigrum*, and should be explained by the fact that *A. thaliana* is diploid and *P. nigrum* is tetraploid. 

**Table 3:** Characterization of proteome predicted from CDS of root libraries

	**Proteins number**	
*Arabidopsis thaliana* root library 5536	8004	
Black pepper root (our work)	4472	
	OrthoMCL proteins families	
Arabidopsis thaliana root library 5536	Orthogroups	3408
	Paralogs	37
	No group	137
Black pepper root (our work)	Orthogroups	1017
	Paralogs	159
	No group	68

### Detection of microsatellites

Microsatellites were identified using the MISA script (http://pgrc.ipk-gatersleben.de/misa/). Analyzing the 22363 contigs, 168 di-, tri- and tetranucleotide repeats were identified, representing 43%, 56% and 1% of the total, respectively (Table 4). The most commonly detected SSR was the trinucleotide AGC, with ACT and ACG the second most common.

**Table 4:** SSR statistics

**Dinucleotide repeat**	**number**	**%**
AC	21	29
AG	44	60
AT	5	7
CG	3	4
Total	73	100
Trinucleotide repeat	number	%
AAC	7	7
AAG	7	7
AAT	8	9
ACC	1	1
ACG	15	16
ACT	15	16
AGC	18	19
AGG	6	6
AGT	10	10
CCG	7	7
Total	94	100
Tetranucleotide repeat	number	
AAAT/ATTT	1	

## Discussion

Biotechnological methods are being successfully employed in plant breeding [16] to confer, for example, increased resistances to pathogens. To use biotechnology in breeding programs, however, prior genetic and genomic knowledge is needed to isolate genes and characterize genotypes and phenotypes [16]. Plant genomics, especially for non-model plants, is always a challenge due to the high levels of ploidy, large genome sizes, low genome complexities and large proportions of repeat sequences. Instead of sequencing the complete genome, sequencing the transcriptome is a good alternative for rapidly and efficiently accessing the expressed genes and for characterizing phenotypes [17].

The characterization of transcriptomes can use hybrid- or sequenced-based technologies, but all hybrid-based technologies require prior knowledge of the transcriptome. In plant biology, Expressed Sequence Tags (ESTs) based on the sequencing of cDNA libraries have been a great advance and have contributed significantly to the study of plant genomics. The sequencing of cDNA libraries, however, has many limitations, such as the cost in time and money involved in cloning and Sanger sequencing. The serial analysis of gene expression (SAGE) and the improved variant SuperSAGE have been used to increase throughput and sampling, but the reliability of the identification of transcripts is one of their limitations [18]. The second generation of sequencing (2-GS) technology circumvents these limitations and generates a large number of reads or datasets of short reads in a shorter time and with a lower reagent cost per base than Sanger sequencing. 2-GS technologies have opened great prospectives for genetic studies of non-model plants [17].

The genome of black pepper is very poorly characterized, with only 184 sequences deposited in Genbank (access 3/11/2011). Previous genetic studies have focused on the diversity, phylogeny and taxonomy of cultivated black pepper and its wild relatives [19-22]. Despite the agricultural and economic importance of this spice, only DNA sequences of phylogenetics markers are available in molecular databases (Figure 1). Another difficulty for the genomic study of black pepper is the lack of a sequenced genome from the Magnoliid group, describe as basal angiosperms group [23,24] (Figure 1). All full-sequenced genomes available in databases are from monocot or dicot plants, plants phylogenetically distant from black pepper, as show in Figure 1[23,24].

About 20 Gb of the data can be mapped, corresponding to 71 million short reads of 50 bp. *De novo* assembly generated 22363 contigs, 10338 unigenes with an N50 of 168 bp. The N50 is slightly less than the 202 bp obtained in an Illumina 75 bp dataset for the *Ipomoea batatas* transcriptome and the 208 bp obtained in a 75 bp single- and paired-end dataset from *Camellia sinensis*[25,26]. The number of contigs, however, is lower than in these other studies: 56516 and 127094 contigs for *I. batatas* and *C. sinensis,* respectively. Although that the read number is higher in our study, (71 million vs 59 and 35 million for *I. batatas* and *C. sinensis)*, the contig number is inferior. This result could be explain by the read lengths were different (50 bp vs 75 pb) and SOLiD dataset is lower in quality, only 50–60% of reads mapped against a transcriptome reference [27,28].

Estimating the amount of coverage of the transcriptome is complex due to the lack of genetic data from black pepper. Black pepper is tetraploid with 2n = 52. The size of its genome, estimated by cytogenetic studies, is about 1220 Mbp (1C = 1.25 pg), which is about ten-fold larger than the genome of *Arabidopsis thaliana*[29]. In *A. thaliana*, about 6% of the genome is transcribed, representing 41671 transcripts [18]. In the *A. thaliana* TGI database, however, two root-derived cDNA libraries have largely been sequenced and have identified 5609 and 5884 transcripts (11.3 and 9.3 Mbp), which seems to indicate that only 14% of predicted genes are expressed in the root [30]. Assuming a similar proportion of transcription in *P. nigrum*, the entire transcriptome is estimated to be 73.2 Mbp (6%) and the root transcriptome to be 10.3 Mbp (0.84%).

The cDNA library was sequenced with a high coverage per base, 62x on average, summing 3.8 Mbp. Shown as an example in Figure 4, the transcript NODE-882-2-0_930 has a good read coverage, up to 20x, and shows a high identity and a good co-linearity with an mRNA sequence from *P. trichocarpa*, indicating a good quality of sequencing and assembly. The comparison of predicted CDSs with the proteomes of other plants demonstrates that the percentage of sequences with significant homology is higher with dicots (51–53%) than with monocots (15–23.4%). This low degree of homology may be due to the phylogenetic distance between magnoliids and others groups (eucots and dicots) and to the different average transcript size (211 pb), as described during the sequencing of the *I. batatas* transcriptome [25]. The method of k-mer additive assembly is efficient in maximizing the number of transcripts, and interestingly, the proportion of transcripts with significant homology (22–35%), the N50 (129 to 236 bp) and the average length of transcript (156–214 bp) are relatively well conserved for each k-mer (19–43 bp) (Figure 2). Comparison with other studies of plant transcriptomes is difficult because the read length used with the Illumina platform is longer (75 bp) and the library is paired-end, which clearly facilitates the process of assembly, as demonstrated for *I. batatas*[25]. Finally, limitations of the 2-GS platform produce high numbers of short contigs, but our dataset has 2144 unigenes over 200 bp in length and about 72% of reads with a significant homology, which represent a significant advance in our biological knowledge of black pepper.

The functional annotation of the dataset of transcripts is informative for the physiology of stems and roots of black pepper. NGS datasets from plant root tissue are only available for ginseng and sweetpotato. The “response to stimulus” and “localization” categories of Figure 5 are highly represented in the transcriptomes from roots of both sweetpotato and black pepper [25]. The profile of molecular functions is conserved between the two root plants. In both, the function “transporter activity” is quite high. This feature may be explained by the fact that roots are very important in the absorption of microelements. This preliminary annotation of the transcriptome from black pepper is very important and should lead to the identification of new genes coding for transporters, transcription factors specific to root and stem tissue or proteins used for defence.

## Conclusion

In model plants, NGS has increased the ability to generate and analyze the expression and detection of most transcripts, to identify genes and their forms of alternative splicing and to analyze DNA methylation and histone modifications on a genomic scale [6,8]. The technology based on Sanger sequencing has generated much data from model species, and plants with sequenced genomes and transcriptomes have provided a robust database reflecting gene expression. The transcriptomic resources for non-model plants, particularly with NGS, however, are limited, especially for the analysis of expressed sequences, alternative splicing and SNPs.

The sequencing of *P. nigrum* using the SOLiD platform (short-reads) produced the first dataset of sequences for a non-model species belonging to the Magnoliid group and order Piperales. Assembly without a reference genome or transcriptome is considered difficult, but a partial transcriptome of the non-model species *P. nigrum* was assembled. This work was aided by the use of some *de novo* assemblies performed using NGS data from non-model plants such as chickpea *Cicer arietinum* L. [31], *Eucalyptus grandis* x *Eucalyptus urophylla* hybrid [32], rubber *Hevea brasiliensis*[33], sweetpotato *Ipomoea batatas*[25] and buckwheat *Fagopyrum*[34].

Analysis of SSRs in the data generated has great potential for applications in studies of genetic diversity, plant breeding and reproduction. The sequences produced in this study are very relevant, since little information about black pepper is available in the biological databases, and the data produced can be used in ecological studies and biotechnology.

## Methods

### Plant material

The tissue samples were obtained from basal root region of eight 70-day-old plantlets. All plantlets used in this study are from Brazilian cultivar of *P. nigrum*, Bragantina ( Additional file 3: Figure S3). The Bragantina cultivar is an ecotype of the Asian Panniyur-1 cultivar, which is a hybrid of the Uthirankotta and Cheriyakaniyakadan cultivars. Stakes obtained from black pepper plants were rooted in trays containing sand and grown in pots containing vermiculite previously autoclaved twice. The plantlets used in this study were grown and acclimatized in a greenhouse for about around two months.

### cDNA preparation and sequencing

Total RNA was extracted from 35 mg of tissue from the stem-root region of *P. nigrum* using the Illustra RNAspin Mini Kit (GE Healthcare, USA). To remove any DNA contamination, the sample was cleaned with the Oligotex Direct mRNA Mini Kit (Quiagen, USA). The rRNAs were depleted using a RiboMinus Eukaryote Kit for RNA-Seq and a RiboMinus Plant Kit for RNA-Seq (Invitrogen, USA). The concentration of the mRNA was determined using a Qubit analyzer (Invitrogen, USA).

An aliquote (126.5 ng/μL) of depleted RNA was fragmented and used to generate one cDNA library for high-throughput sequencing. The library of fragments was obtained using the SOLiD Total RNA-Seq Kit (Invitrogen, USA). The global procedure of this kit is based on the hybridization of adapters with degenerate ends, followed by reverse transcription and library amplification by PCR [35]. The cDNAs were selected by size on a polyacrylamide gel before and after the library amplification. The sequencing of the cDNA library was performed in a well of a flow cell.

### Pre-processing sequencing data

The SOLiD adapter sequences were trimmed from reads using CutAdapt (0.9.5) [36], and the sequences of reads were converted from SOLiD encoding color space into double-encoded colors in FASTQ format. PRINSEQ (lite 0.14.4) [37] was used to remove redundant reads, to trim low quality reads (average QV < 20 for a 3-nt window, and QV < 18) and to remove small pre-processed reads (read length < 30 bp) [37]. Statistics of the dataset of reads was obtained using FastQC software (V. 0.9.2) (http://www.bioinformatics.bbsrc.ac.uk/projects/fastqc/).

#### *De novo* transcriptome assembly and functional annotation

Transcriptome assembly was performed using Velvet and Oases [38,39]. For optimizing the *de novo* assembly, the method of additive multiple-k [12] was used to combine the properties of multiple assemblies using different k-mers (19–43).

The program ESTScan was used to detect potential coding regions in the transcript sequences obtained by assembly [40]. In order to obtain the protein sequence the FrameD tool was employed [41]. The program BWA [42] was used to align raw reads against all contigs obtained by Velvet assembly for calculating the coverage of the sequencing.

Local BLASTX (E-value 1e^-05^) was used to search homologous sequences against plant databases from PlantGDB (ftp://ftp.plangdb.org), nr-viridiplantae (NCBI), *V. vinifera*, *A. thaliana*, *P. trichocarpa*, *G. max* and *O. sativa*.

The functional annotation was based on BLASTX results with the nr-viridiplantae database, and the program BLAST2GO was used to assign biological functions, cellular components and cellular processes to the transcripts [43]. The groups of orthologous and paralogous proteins were predicted using orthoMCL tools [44].

### Detection of microsatellites

The MISA perl script was used to identify the position of SSRs in the sequence dataset (http://pgrc.ipk-gatersleben.de/misa/misa.html). The search for SSRs in our transcriptome was performed using the detection of bi-, tri- and tetranucleotide repeats, present more than 5x and with a minimum distance of 100 bp.

## Competing interests

The authors declare that they have no competing interests.

## Authors' contributions

Conceived and designed the experiments: SMCG, ECOM, SMR, AS, HS, WASJr, MICS, SD. Performed the experiments: SMCG, DGP, ECOM, SMR, MCP, ORFL, IT, RTJR, SD. Contributed reagents/materials/analysis tools: SMR, AS, HS, WASJr, MICS. Wrote the paper: SMCG, AS, HS, WASJr, MICS, SD. All authors read and approved the final manuscript.

## Supplementary Material

Additional file 1 **Figure S1.** Statistical analysis of quality value (QV) of sequencing dataset. The quartiles, median and average were plotted for each position of the dataset of reads. Click here for file

Additional file 2 **Figure S2. **Size distribution of the filtered and trimmed reads from 2-GS sequencing from the black pepper root. Click here for file

Additional file 3 **Figure S3. **Picture of black pepper root used for cDNA library. A) Global view of 70 day old plantlets. The red box indicates the root region used to extract RNAs. B) Sectional view of black pepper root at the region used to extract RNAs. Click here for file
