# Supplementary figures and images for: High-throughput sequencing of black pepper root transcriptome
Source: BMC Plant Biol. 2012 Sep 17;12:168. doi: 10.1186/1471-2229-12-168 (PMC3487918; doi:10.1186/1471-2229-12-168)

Figure S1.

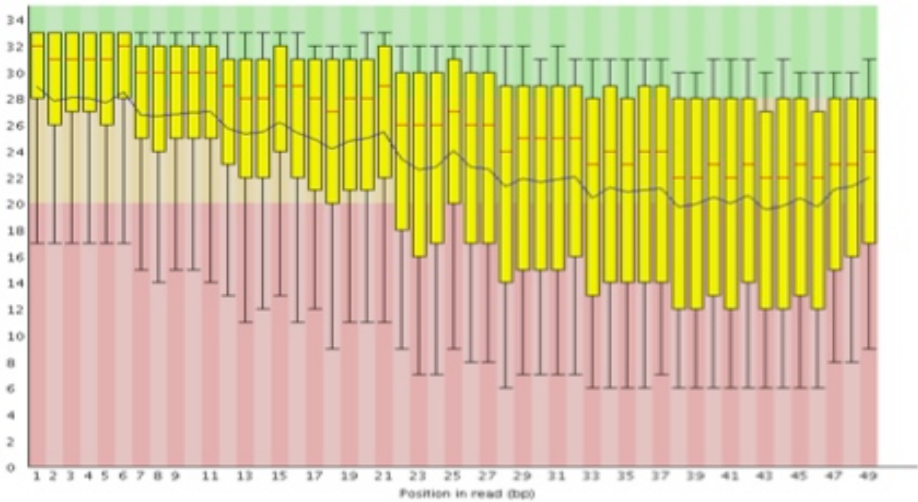

Supplement: Additional file 1 — Figure S1. Statistical analysis of quality value (QV) of sequencing dataset. The quartiles, median and average were plotted for each position of the dataset of reads. [file 1471-2229-12-168-S1.pdf]

Figure S2

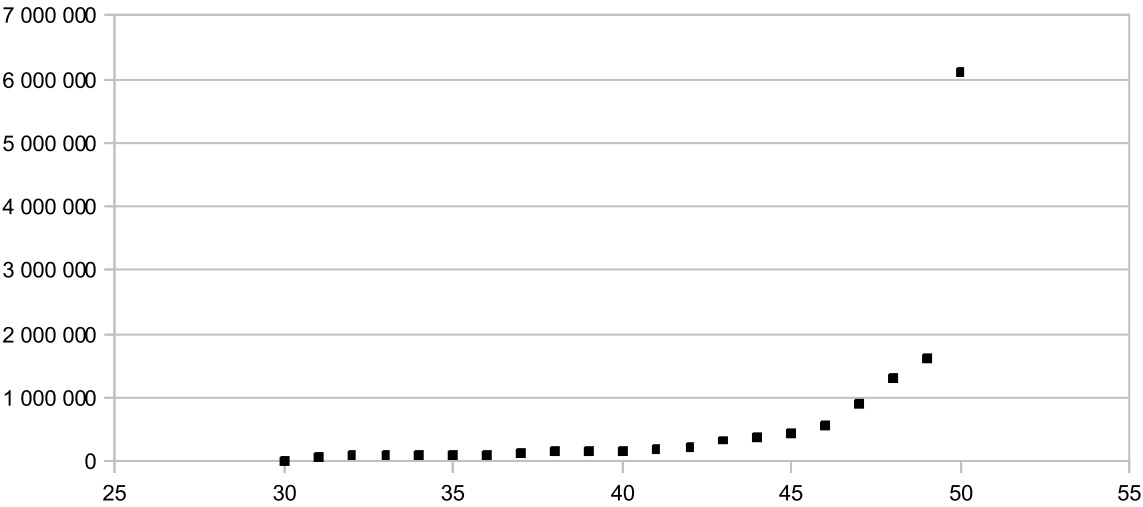

Supplement: Additional file 2 — Figure S2. Size distribution of the filtered and trimmed reads from 2-GS sequencing from the black pepper root. [file 1471-2229-12-168-S2.pdf]

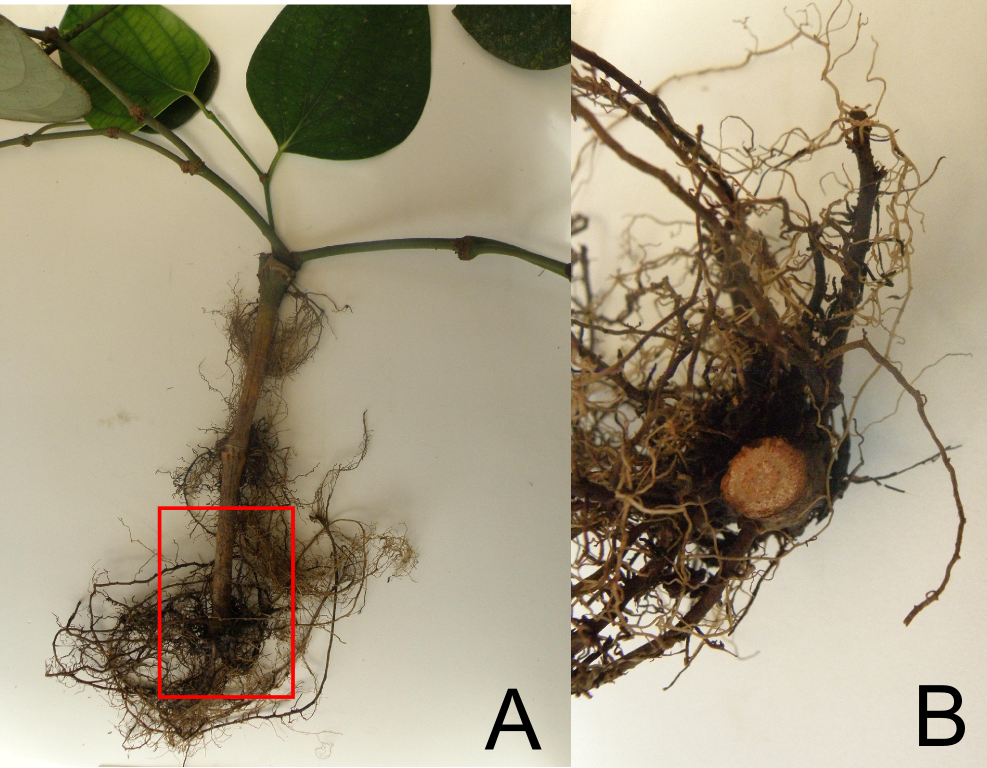

Supplement: Additional file 3 — Figure S3. Picture of black pepper root used for cDNA library. A) Global view of 70 day old plantlets. The red box indicates the root region used to extract RNAs. B) Sectional view of black pepper root at the region used to extract RNAs. [file 1471-2229-12-168-S3.png]
